# Emotional intelligence as a contributor to enhancing educators’ quality of life in the COVID-19 era

**DOI:** 10.3389/fpsyg.2022.921343

**Published:** 2022-08-22

**Authors:** Prashanti Maharaj, Anisha Ramsaroop

**Affiliations:** School of Management, Information Technology and Governance, College of Law and Management Studies, University of KwaZulu-Natal, Durban, South Africa

**Keywords:** COVID-19, educators, emotional intelligence, quality of life, resilience

## Abstract

The basic education fraternity is constantly evolving with various stressors among others, curricular changes, adaptation to the Fourth Industrial Revolution, poor educator development, excessive workload, and brain drain, thus negatively affecting educators’ quality of life. The Coronavirus (COVID-19) has expedited the importance of emotional intelligence, as an essential resilience skill for enhancing the quality of life during adversity. The objective of the study is to ascertain the relationship between emotional intelligence and the quality of life of educators. A quantitative approach was utilized using simple random sampling. A sample of 108 educators from a population of 154 was drawn from six schools in the Reservoir Hills precinct of KwaZulu-Natal. The findings revealed a significant relationship between emotional intelligence and the quality of life of educators. A practical research model was advocated for key stakeholders in the South African basic education sector.

## Introduction

The Future of Jobs Report by the World Economic Forum (2020) ranks emotional intelligence as one of the top 15 in-demand skills required to thrive in 2022 and beyond. Three sole theorists have evolved the concept of emotional intelligence, namely: Mayer and Salovey (1990), Goleman (1998), and Bar-On (2006). Goleman (1998, p. 137) defines emotional intelligence as the “capacity for recognizing our own feelings and those of others, for motivating ourselves, and for managing emotions well in ourselves and in our relationships.” However, Bar-On (2006) argues that emotional intelligence is a multifactorial set of competencies, skills, and facilitators that determine how people express and understand themselves, understand and relate to others, and respond to daily situations. The Ability Model by Mayer and Salovey (1990) who are the pioneer researchers of the construct emotional intelligence underpins this study. The Ability Model proposes that emotional intelligence entails the following abilities: appraisal and expression of emotion: the ability to show and to understand own and others’ verbal and non-verbal emotion expressions, regulation of emotion: the ability to control own and others’ emotions, and the utilization of emotion: the ability to use emotion to enhance flexibility, creativity, and motivation by facilitating logical thinking processes. The model posits that emotionally intelligent people can feel emotions of the self and others and use that knowledge to influence and change the environment thus enhancing resilience and quality of life (Mayer and Salovey, 1990; Katungu, 2018). Therefore, in a school setting, emotional intelligence will equip educators to manage daily stressors with patience, insight, and innovation, as well as to navigate interpersonal interactions with empathy (Park and Rhee, 2020). Drigas and Papoutsi (2020), advocate that people in the face of a crisis tend to stand together and cooperate because people understand that the risk is shared and are worried not only about themselves but also about others, which helps foster resilience in themselves and in others. Therefore, McDonald (2021) emphasizes that high emotional intelligence enhances adaptive regulation of distressing emotions as well as successful management of daily stressors and challenges.

Zeidner et al. (2012, p. 54) describe emotional intelligence as the “ability to identify and manage your own emotions and the emotions of others.” Quality of life can be described as the “individuals’ perception of individuals’ positions in life, in the context of the culture and value systems in which they live and in relation to their goals, expectations, standards and concerns” (Pinto et al., 2017, p. 7). Pavliscak (2018) confirms emotional intelligence is a gateway to a balanced-life as it is essential to every quality of life component. Studies therefore indicate that emotional intelligence can enhance the quality of life, because people feel a sense of well-being when one’s work and lives are meaningful (Hsiang, 2016; Anjum and Swathi, 2017; Koçak, 2021). The role of the educator continues to be a challenge (Carroll et al., 2022). Educators encounter not only heavy workloads and time constraints in teaching, but also student discipline and behavioral difficulties, pressure from insensitive administrations, ongoing curricular challenges, work–life balance challenges, and an overload of responsibilities (Mapfumo et al., 2012; Mukwamu, 2019; Velle, 2020; Sindhya, 2022). This environment creates psychological and physical ill-health, as well as emotional distress in educators (Mohamad and Jais, 2016; Sindhya, 2022). Therefore, by indoctrinating emotional intelligence in educators, educators will have the ability to cope with such insurmountable demands by being able to motivate oneself and persist in the face of frustrations, control impulse and delay gratification, regulate one’s mood, minimize distress from the overwhelming the ability to think; and establishing empathy (Goleman, 2017). As a result, emotional intelligence promotes enhanced quality of life, as emotional intelligence assists the individual to cope with life situations, and understanding emotional intelligence within multiple perspectives (Al-Huwailah, 2017). Studies have confirmed that emotional intelligence increases with age, being married and having high educational levels (Madahi et al., 2013; Kanesan and Fauzan, 2019; Addae and Ofosuhene-Mensah, 2021). Therefore, individuals with high emotional intelligence will have an enhanced quality of life. This is as a result of emotional intelligence having a positive relationship with the quality of life (Keefer et al., 2018; Pavliscak, 2018).

The 2011 State of the Heart Report by Six Seconds Emotional Intelligence network has been tracking global developments in emotional intelligence (Freedman, 2018). The network is the world’s most comprehensive research on emotional intelligence strengths, challenges, and possibilities. After surveying 200,000 people in 160 countries, the findings give significant insights on the shifting capabilities to construct a better future, indicating important trends for global emotional intelligence comprising personal well-being, effectiveness, relationships, and quality of life (Freedman, 2018). The value of emotional intelligence in Africa, Asia Pacific, Europe, Latin America, the Middle East, and North America was determined by comparing national-average emotional intelligence scores to World Health Organization data on well-being (Freedman, 2018). It was observed that the majority of Asia Pacific and Middle Eastern countries had low well-being and emotional intelligence (Freedman, 2018). Furthermore, Africa was viewed as having the potential for high emotional intelligence to improve well-being (Freedman, 2018). Teaching in primary, secondary and special needs schools is widely regarded as one of the most stressful occupations in the world because it demands empathy, love and affection for students (Viac and Fraser, 2020). An educator is an emotional laborer who must continuously cope with ascertaining emotional intelligence. According to Kant and Shanker (2021), educators are supposed to exhibit positive emotions at all times, regardless of what is going on in their inner cognition.

Throughout the years, South Africa’s basic educational system underwent a variety of curriculum changes, namely Curriculum 2005 was introduced in 1997, Revised National Curriculum Statement introduced in 2002, National Curriculum Statement introduced in 2007, and Curriculum and Assessment Policy Statements was introduced in 2012 (Adu and Ngibe, 2014). The Minister of Basic Education, Angie Motshekga stressed that the education system would have to be overhauled to meet the demands of the 4IR (Davis, 2018). As a result, President Cyril Ramaphosa confirms that a draft coding and robotics curriculum for schools would be gazetted in the course of 2021 and rolled out in 2023 (South African Government News Agency, 2021; Mndende, 2022). Adu and Ngibe (2014) affirm that constant changes in the curriculum affect effective teaching and learning because these changes present a challenge to educators to reach the expected student performance. Molapo and Pillay (2018) posit that educators do not feel well-equipped to implement new curriculum changes because most educator-training is offered as short-term programs involving several hours or days of workshops. Govender (2018) explains that challenges exist in the design of sustainable and ongoing activities for professional development, related to lessons in the classroom that improve teaching and focus on educators’ needs, rather than reliance on once-off workshops.

A London-based publication, The Economist, stated that South Africa has one of the worst education systems due to the poor quality of education, with one of the factors being the lowered pass rate requirements actioned by the Department of Basic Education (Mukwamu, 2019). Allan Thompson Deputy Chairperson of the National Teachers’ Union, mentioned that instead of lowering the pass mark, there should be an increase in educators in the classroom and a reduction in the class size (Nkosi, 2018). However, it is not possible to have an increase of educators in the classroom due to the challenging working conditions, lack of resources and budgets (Du Plessis, 2020). Challenges have an adverse effect on educators’ quality of life resulting in a high attrition rate (Miya, 2017; Niekerk, 2017). Govender (2018) discovered that countries such as the United Arab Emirates were offering better working conditions and support to South African educators (Govender, 2018). This has consequences for the brain drain thus leaving South Africa with a lack of valuable and experienced educators (Govender, 2018). Educators’ emotions are heightened adversely as educators have limited support mechanisms for coping and managing challenges (Bearschank, 2010; Navsaria et al., 2011; Nel et al., 2016). For example, South Africa has undergone a number of curriculum changes, but educators still find it difficult to transition smoothly (Phakgadi, 2018).

Such challenges have now been exacerbated by a new set of challenges brought on by the Coronavirus (COVID-19), which has altered all aspects of educators’ quality of life. Educators were summoned to support students’ academic development and well-being throughout this continuous transition, while also navigating adversity in their own lives provoked the inability to manage stress, anxiety and fear (Niekerk and Gent, 2021; Sindhya, 2022). The University of KwaZulu-Natal’s Professor Anja Philipp advocates that the profession of the educator has evolved dramatically to support remote teaching and learning while progressively returning to contact teaching (Ishmail, 2020). Additionally, educators are managing “techno-stress” that is caused by the introduction of technology thus posing psychosomatic consequences with the development of high levels of burnout (Muñoz et al., 2020). Research indicates several challenges such as, adaptation to online teaching, economic uncertainty, educators being pressured to deliver the school curriculum timeously, fear of health and safety caused by the pandemic, educators being blamed for poor performance, the inability to read students verbal and non-verbal cues with a mask on, and the challenge for students to understand educators verbal and non-verbal cues, and managing work–life balance, exist (Allen et al., 2020; Pokhrel and Chhetri, 2021; Sindhya, 2022). These stressors have created contentious distressing emotions and burnout, hence negatively impacting educators’ overall well-being (Ishmail, 2020).

There has been little evidence in the education fraternity to establish the critical significance of emotional intelligence in enhancing educators’ quality of life, particularly in the current setting of the pandemic, where emotional intelligence is paramount. This study therefore has relevance and value and contributes to enhancing well-being from a global South perspective. Bhuvaneswari and Baskaran (2020) and Uniyal and Rawat (2020) agree that there is limited research; hence emotional intelligence in a school setting is needed. The need for emotional intelligence has been popularized by COVID-19, and it will become increasingly vital in the post-COVID-19 period as work becomes more flexible, more remote and ambiguous (Drigas and Papoutsi, 2020; Uniyal and Rawat, 2020). This is the first time that the education fraternity in South Africa experienced a sudden life-changing catastrophe which will soon become the largest pandemic of the 21st century in a form of a natural disaster (Schroder et al., 2021). Therefore, this research is valuable for South African educators to acquire skills to appraise, regulate, and utilize their emotions positively, as this will promote resilience strategies to help maintain educator’s physical, social, psychological, and environmental well-being.

From a global South perspective, with Africa being the focus, there is a potential for high emotional intelligence to improve well-being (Freedman, 2018). Abdel-Fattah (2020) highlights that COVID-19 has expedited the need to understand the role of emotional intelligence when confronted with adversity. This study advocates that COVID-19 has caused disruptive innovation with the need for creativity to flourish through devising mindful strategies for enhancing educator quality of life during COVID-19 which is reiterated in the practical research model (Appendix G). Creativity is established through emotional intelligence enabling positive emotions (Goleman, 2017). One of the focus areas that emerged at the 10th African Confederation of Principals Conference held in 2018 was the use of emotional intelligence, which will aid in revolutionizing the teaching profession in Africa (Association for the Development of Education in Africa, 2018). Furthermore, Yale President and one of the founders of the concept of emotional intelligence, Peter Salovey, who spoke at Lagos Business School in Nigeria for Yale Africa, emphasized the need to develop emotional intelligence skills for thriving in life (Lagos Business School, 2020).

The concept of quality of life assumes that a job is more than just a job because it is the core of a person’s life (Sanchez et al., 2019). Quality of life encompasses three facets. Firstly, physical health, comprising pain, discomfort, work capacity, and dependence on medicinal substances and aids, sleep, and rest. Secondly, psychological health, which entails negative and positive feelings, body image, self-esteem and concentration; social relationships evolving into personal relationships and social support. Lastly, environmental health, which constitutes financial resources, freedom, home environment, health and social care, physical safety, security, and the physical environment” (Dubey, 2012, p. 64). Educators who have a high quality of work life are more likely to report higher levels of job motivation and a greater commitment to remaining in the profession (Viac and Fraser, 2020). Pre-COVID-19, educators across the world were already experiencing a low quality of life perception, with a major impact on psychological and physical health owing to numerous stress factors linked to work overload (Lizana et al., 2021). Research indicates that stressful working conditions can cause occupational illness, resulting in a low quality of life that impacts educators’ motivation, self-efficacy, and job commitment (Toropova et al., 2020). Viac and Fraser (2020), argue that educators who feel supported by their colleagues and principals in the school setting during the pandemic have a higher sense of quality of life because they have greater self-efficacy, less work pressure, and are more pupil-orientated, making educators better equipped to deal with external pressures. The psychological well-being of educators in a time of COVID-19 has become an increasing concern not only in South Africa, but also across the world with educators feeling fearful that the pandemic will worsen (Mahamba, 2021). Psychological health of educators is often neglected and has been exacerbated by COVID-19 (Niekerk and Gent, 2021). The United Nations (2020) observed an increased rate of stress and anxiety being the most cited reaction during the pandemic.

Emotional intelligence increases an educator’s quality of life by assisting in many areas such as the educator being less impulsive, less controlling, the ability to manage stress, increased self-assertiveness, expression of emotion, maintaining positive outlooks, making better judgments, improved communication, positively influencing people, enhanced work–life balance, and being emotionally resilient (Mustafa et al., 2020). However, having high emotional intelligence does not mean that a person will not suffer stress or anxiety over everyday difficulties, much less a more stressful event like COVID-19 (Drigas and Papoutsi, 2020). Rather, the individual will be conscious of his/her circumstances and the feelings he/she is experiencing by employing resilience techniques in order to have self-control, self-management, and not be deceived by his/her anxiety, with serious physical and psychological effects (Drigas and Papoutsi, 2020).

## Materials and methods

The objectives of the study are as follows:

- To measure the level of emotional intelligence of educators.
- To measure the level of the quality of life of educators.
- To examine the relationship between emotional intelligence and the quality of life.
- To establish if there is a significant difference on each biographical variable (gender, age, race, marital status, education, number of years in the teaching profession, level currently teaching, and employment status) on the quality of life and emotional intelligence of educators.

### Participants and procedure

A descriptive design with a quantitative research approach was used to provide adequate data to test the relationship between emotional intelligence and the quality of life. The study site consisted of primary, secondary, and special needs schools. The target population adopted in this study was permanent and part-time school educators, from six schools. The sample design adopted was simple random sampling and was chosen using a population list that was provided by the six school administrations. The population was numbered consecutively, and thereafter, participants were selected from the list by means of a computer-generated method. The total population size of educators being 154, the sample represented 108 educators, mostly females (82.4%) with the dominant race being Indian (82.4%). The majority of respondents were between the ages of 20 and 29 with 54.6% married, 96.3% employed permanently, 33.4% in possession of an undergraduate degree, 31.5% teaching at junior level, and 51.9% with a length of service more than 10 years. Reasons for non-participation by all educators were not made known to the researchers. Non-participation could have been attributed to several reasons, among others, non-availability of educators or a disinterest in the study.

### Measures

Close-ended personally administered questionnaires were utilized. Biographical details of participants were elicited using nominal and ordinal scaling.

Emotional intelligence was assessed using the Schutte Self-Report Emotional Intelligence Test on a five-point Likert scale (Schutte et al., 2009). The Schutte Self-Report Emotional Intelligence Test is a 12-item self-report questionnaire that comprises three sub-scales: appraisal of emotions (four items—“I know why my emotions change”), regulation of emotion (four items—“I seek out activities that make me happy”), and utilization of emotion (four items—“I am aware of the non-verbal messages other people send”).

The World Health Organization Quality of Life Assessment on a five-point Likert scale (The Whoqol Group, 1998). It consisted of 12-item that measures the following sub-dimensions: physical health (three items—“I am satisfied with my capacity for work”), psychological health (three items—“I often have negative feelings such as blue mood, despair, anxiety, and depression”), social relationships (three items—“I am satisfied with the support I get from my friends”), and environmental health (three items—“My physical environment is healthy”).

Appendix A reflects the Cronbach’s Coefficient Alpha score for all the individual items that constituted the questionnaire.

### Data analysis

Descriptive and inferential statistics were utilized in the study. Frequencies and percentages were used for interpreting biographical data. Measures of central tendency were used to help the researcher interpret the characteristics of the sample, and finally measures of dispersion was used to help measure how spread out a set of data is. Chi-square tests were performed to determine if the scoring patterns per statement had a statistical difference between the nominal (gender, race, marital status, and employment status) and ordinal (highest level of completed education, number of years teaching, level currently teaching, and age) biographical variables.

Pearson’s Correlation Coefficient was used to measure the statistical relationship and strength between emotional intelligence and the quality of life. A principle component factor analysis was used as the extraction method with the rotation method being Kaiser–Meyer–Olkin and Orthogonal Varimax Rotation. All of the conditions were met for factor analysis. That is, the Kaiser–Meyer–Olkin Measure of Sampling Adequacy value should be greater than 0.500 and the Bartlett’s Test of Sphericity sig. Value should be less than 0.05. Factor analysis was done for Likert scale items resulting in certain components divided into finer components. Appendix B illustrates the results and showcases the inter-correlations between variables.

Cronbach’s Coefficient Alpha measured at an overall score of 0.798 for the 22 items that constituted the questionnaire. The results indicate acceptable high internal consistency, that is, how closely related a set of items are as a group (Sekaran and Bougie, 2016).

## Results

Data collected from the responses were analyzed with Statistical Package for Social Sciences version 25.0.

### Objective 1

Scoring patterns of the respondents were analyzed per variable per section. The results in Appendix C are presented using summarized percentages for the sub-dimensions of emotional intelligence against each statement. The following patterns were observed:

- all of the statements show significantly higher levels of agreement;
- there are no statements with higher levels of disagreement; and
- chi-square *p* < 0.05 values imply that the differences between how respondents scored were significant.

Applying emotional intelligence data pertaining to educators indicates that those with high emotional intelligence are more empathetic and better at fostering a learning environment that supports the development of students’ socio-emotional skills, hence enhancing resilience for educators’ quality of life (Huang and Xu, 2019; Cristóvão et al., 2020; McDonald, 2021). Therefore, if an educator fosters emotional intelligence, emotional intelligence can lead to a fulfilling life (Abiodullah et al., 2020). Educators in the present study exhibit high levels of emotional intelligence, implying that they have the ability to appraise, regulate, and utilize their own emotional competencies, enabling them to cope with stressors and adversities, thereby improving resilience in their personal and professional lives. Objective 1 is thus achieved.

### Objective 2

Scoring patterns of the respondents were analyzed per variable per section. The results in Appendix D are presented using summarized percentages for the dimensions of quality of life against each statement. The following patterns were observed:

- all of the statements show significantly higher levels of agreement;
- there are no statements with higher levels of disagreement; and,
- chi-square *p* < 0.05 values imply that the differences between how respondents scored were significant.

Stressful working conditions can cause occupational illness, resulting in a low quality of life that impacts educators’ motivation, self-efficacy, and job commitment (Toropova et al., 2020). Furthermore, the frequency and quality of relationships with others (that is, the school community, family, friends, and peers) can have a favorable or unfavorable influence on educators’ quality of life. This is supported by Viac and Fraser (2020), who argue that educators who feel supported by their colleagues and principals in the school setting have a higher quality of life because there is greater self-efficacy, less work pressure, and a more pupil-centered orientation, making them better equipped to deal with external pressures. Educators in the current study have a high level of quality of life, indicating that adversity showcases vulnerability such as looking to loved ones for help and emotional support, utilizing social media platforms for engagement, turning to spirituality and mindfulness, increasing one’s self-care, and focusing on aspects of the situation that are under the individual’s control, for example, refraining from exposing oneself to propaganda that elicits distressing emotions. Therefore, educators in the study are content with their professional working conditions due to organizational support, and self-developed coping techniques. Objective 2 is therefore achieved.

### Objective 3

Pearson’s Correlation Coefficient presented in Appendix E was performed on the ordinal data. The results in Appendix E indicated all significant relationships by a * representing a 1% level of significance and ** representing a 5% level of significance.

Emotionally intelligent educators are capable of balancing emotional demands (Kant and Shanker, 2021). Prior to the pandemic, educators frequently experienced loneliness, a lack of motivation, burnout, emotional stress, and distressing emotions which have further been exacerbated by COVID-19 (Ishmail, 2020; Zhang et al., 2022). Pioneer theorists Mayer and Salovey (1990) confirm that appraising and expressing emotions in oneself and others, regulating emotions in oneself and others, and utilizing emotions all contribute to determining coping behaviors and subsequent adaptive outcomes, hence improving one’s quality of life (Keefer et al., 2018). Appendix E thus confirms a significant relationship between emotional intelligence (sub-dimensions being appraisal, regulation, and utilization) and quality of life (encompassing sub-dimensions such as physical health, psychological health, social relationships, and environmental health) of school educators at a 1 and 5% level of significance. This implies that educators in the study utilize their emotional intelligence abilities as a resilience tool to assess stressful situations generally and advocate customized coping behaviors that will protect their quality of life from deteriorating. Hence, objective 3 is achieved.

### Objective 4

A second chi-square test was performed to determine whether there was a statistically significant difference between the nominal and ordinal variables. Appendix F indicates that there is a significant difference between gender, race, marital status, education, number of years in the teaching profession, level currently teaching, and employment status influencing emotional intelligence and quality life of educators (*p* < 0.05). Notably, employment status followed by highest level of education and marital status yielded the most significant differences. Uniyal and Rawat (2020) argue that people who are successful in controlling their stress levels in spite of an increased workload, have stronger emotional intelligence, which improves their quality of life.

In relation to employment status, when educators are pursuing a profound purpose or engaging in work that is personally important, educators experience significant positive effects (Fourie and Deacon, 2015; Whittington et al., 2017). Positive outcomes include increased levels of commitment, empowerment, satisfaction, and a sense of fulfillment (Davis, 2017; Whittington et al., 2017). As a result, meaningful work leads directly to higher levels of engagement but it also impacts on the levels of employee satisfaction, educator commitment to the organization, and the educator’s willingness to go beyond role expectations to serve others (Rainey, 2014; Martela and Pessi, 2018). Therefore, when work is perceived as meaningful, educators have a sense of fulfillment and purpose that provides a psychological sense of well-being (Geldenhuys et al., 2014; Rainey, 2014; Martela and Pessi, 2018). The experience of meaningful work and well-being contributes to the overall sense of an individual’s life purpose (Geldenhuys et al., 2014; Rainey, 2014; Martela and Pessi, 2018).

Educational qualification is a useful statistic as it indicates that the responses gathered would have been from an informed (learned) source and the distribution of the study’s responses had more educated respondents. Many educators have an honors degree due to their wanting to further their knowledge in the field (Jennifer and Delia, 2018). Additionally, the Department of Basic Education requires future educators to undergo a 4 year Bachelor of Education degree or a 3 or 4 year Bachelor’s degree, followed by a 1 year Postgraduate Certificate in Education (PGCE; Department of Basic Education, 2019).

Teaching as a career allows for many holidays thus posing flexibility (Fransman, 2014; Barik, 2017). This suggests that the career of teaching allows work–life balance for married couples, and partners, especially if these individuals have children (Reddy et al., 2010; Barik, 2017). Educators who are single could imply that the growing demand for educators in the past decade has brought in many younger educators than before, hence young people (possibly university graduates) are more likely to be single than older people (Joan and Henry, 2014; Odanga et al., 2015).

Age, however did not influence educator’s emotional intelligence and quality of life. Mayer and Salovey and Goleman argue that emotional intelligence is ability based and not a trait (that is, it is consistent behavior over time) as it increases by age and training (Kanesan and Fauzan, 2019). Objective 4 is therefore achieved.

## Contribution

From a global south perspective, provinces in South Africa and ultimately Africa have the potential for high emotional intelligence to improve well-being (Freedman, 2018). Therefore, based on the study’s findings and considering the new stressors educators experience, a practical research model (Appendix G) was proposed for key stakeholders in order to continue fueling emotional intelligence in Africa hence enhancing quality of life. Appendix G depicts the variable emotional intelligence with its essential tailor-made strategies demonstrating a significant relationship with the quality of life, which will serve as a resilience mechanism for the South African basic education fraternity to thrive in the face of adversity, like COVID-19. Consequently, when key strategies are executed fully by the relevant stakeholders, it will aid in the development of skills to appraise, understand, manage, regulate, and utilize emotions positively during challenging situations, hence improving the quality of life. A few recommendations among others, stemming from the findings of the study are briefly outlined below:

### Department of basic education

#### Employee assistance programs

Comorbidities remain a critical health risk for educators since educators increase their chance of contracting COVID-19 due to a weaker immune system or the necessity for more care that exposes them to others, hence deteriorating the quality of life (Gillespie, 2021). With educators back to classroom teaching, the executive director of the National Professional Teachers Organisation of South Africa, Basil Manuel, believes that schools must safeguard the well-being of educators, with health and safety regulations so that educators do not become ill (Hlati, 2021). Additionally, UNESCO (2020) confirms that educators who have developed their own psychosocial skills through regular professional debriefing and learning sessions with internal counseling services are better equipped to provide psychosocial support to their students and help themselves and their students navigate the uncertainty and anxiety that the pandemic brings.

The problems caused by the COVID-19 virus are multifaceted. While the resulting concerns generate health, economic, social, and psychological issues, such challenges also trigger each other (Koçak, 2021). Unemployment, isolation, fear, stress, and anxiety of ill-health, all have a negative impact on psychological health during the pandemic (Koçak, 2021). The growing awareness of psychological health and people being more open about it began long before the COVID-19 outbreak, but the current circumstance has accelerated the trend (Singleton, 2020). Qiu et al. (2020) find that fear of insecurity and uncertainty brought on by COVID-19, as well as the separation and loss of crucial relationships and significant lifestyle changes, have elicited strong emotional responses that pose a threat to psychological health. History has showcased that the effect on psychological health from disasters outlasts the physical impact, implying that the present increased psychological health requirement will continue far beyond the COVID-19 outbreak itself (Panchal et al., 2021). As the weight of the crisis begins to lift, health experts predict a long-term impact on people’s psychological health (Joseph, 2021; Koçak, 2021). Considering the adverse effect on psychological health and well-being, the need for the implementation of Employee Assistance Programs (EAPs) are imperative for aiding post-traumatic growth (Lichtman, 2020).

Employee Assistance Programs (EAPs) are designed to provide a variety of psychological services to employees (Baskar et al., 2021). Miller (2019) argues that Employee Assistance Programs (EAPs) are underutilized because employees do not know about them, do not understand what they provide and employees may feel there is a stigma around using these services. COVID-19 has however resulted in an increased focus on Employee Assistance Programs (EAPs). Veldsman and Van Aarde (2021) postulate that employee quality of life and Employee Assistance Programs (EAPs) came under scrutiny as organizations struggled to remain productive while assisting employees in dealing with unprecedented change. Higher levels of stress, anxiety and fear had a significant impact on the workforce, hence Employee Assistance Programs (EAPs) were utilized to provide security and support (Couser et al., 2020). The study recommends organizations design and utilize Employee Assistance Programs (EAPs) which are aligned to organizational culture and practices and the latest well-being trends popularized by COVID-19.

### School leaders

#### Support groups

According to the study, support groups for educators should be formed for educators to connect with their peers and improve educator well-being. Support groups can concentrate on:

- self-care;
- provision of teaching tips and professional development;
- examining strategies and activities for a healthy work–life balance;
- creating work boundaries for what is deemed a healthy work environment, such as ceasing to glorify overworking after hours and instead encouraging rest; and
- identifying trending themes on which educators can share their knowledge and assist one another.

There are numerous examples during the pandemic of educators virtually connecting to support one another through peer, professional and psychosocial support, including mobile coaching and mentoring, even in crisis scenarios. Costa Rica, Croatia, and the Philippines, for example, are leveraging virtual platforms to provide support to educators for professional and personal development (UNESCO, 2020).

### Educators

#### Self-affirmations

Self-affirmations are “self-generated thoughts or experiences that bring about an expanded and positive view of the self and can reduce resistance to situations perceived as threatening” (Hill et al., 2020, p. 1). Self-affirmations can help ease anxiety, mitigate negative emotions, decrease stress, increase well-being, make people more open to behavior change, boosts confidence, and stay on track with recovery goals (Cohen and Sherman, 2014; Hinders, 2020). According to the self-affirmation theory (1988), people are motivated to preserve a positive self-view; therefore, when an individual’s self-competence is endangered, self-affirmations can restore self-competence by allowing individuals to reflect on sources of self-worth being the individual’s core values (Cascio et al., 2016). Self-affirmations have become crucial for coping with COVID-19 stressors. While COVID-19 is a negative circumstance, using affirmations can help one respond and react to one’s emotions in a more positive way (Epton et al., 2020; Van der Veen, 2021). For example: “I believe in my ability to get through tough times,” or “I will not stress over things I cannot control” (Gillman et al., 2022). There are mobile self-affirmation apps, for example, “ThinkUp: Positive Affirmations,” founded by Irit Wald and Jenny Shalev. The app is a leading self-affirmation app that has favorably increased user well-being (Springer et al., 2018; Pangilinan, 2020). The self-affirmation mobile apps are an effect technique to assist users in manifesting positive affirmations, hence improving well-being.

Li et al. (2020) show for the first time that an experimental intervention involving personal value affirmation can buffer psychological stress response during the COVID-19 outbreak. Values consisted of discipline and character, ability and talent, public interest, family and kinship, money and wealth, fame and status. Participants who validated their values, in particular, did not experience increased levels of anxiety as compared to control participants. Furthermore, a spontaneous self-affirmation study was conducted through determining predictors of time spent accessing news about COVID-19, adherence to United Kingdom government recommendations, and worry from COVID-19 related news. Each predictor was regressed onto spontaneous self-affirmation (Epton et al., 2020). Self-affirmation such as “If I am feeling threatened or anxious then I will remind myself of my strengths or values” (Epton et al., 2020). Results indicated that threats arising from the pandemic and the measures used to mitigate it seemed to increase the use of spontaneous self-affirmation in the United Kingdom population which assisted with a change in behavior and stress management (Epton et al., 2020).

## Conclusion

The results of the study confirm that educators possessing high levels of emotional intelligence add value to the different facets of their lives when presented with adversity. The practical research model (Appendix G), generated in the study demonstrates how the dimensions of emotional intelligence (appraisal, regulation, and utilization of emotion) influence each facet of quality of life (physical health, psychological health and environmental health). The positive association of the variables therefore enhances the quality of life of educators thereby allowing educators to develop resilience, to thrive and cope in the post-COVID age, and during adversity.

Several limitations had a bearing on the study. Time constraints were a barrier because it was a lengthy process to gain authorization from the Department of Basic Education, hence causing the study to be delayed. The sample size was inadequate to generalize to all school educators at large. The educators’ time constraints in filling out the questionnaires also impacted the study’s deadline. In terms of recommendations, firstly, for future research, the authors recommend a mixed methods approach in an attempt to determine the richness of the data and to increase validity in the findings. A comparative study using a mixed methods approach between two districts in KwaZulu-Natal to further enhance outcomes is also recommended. The study was located in the province of KwaZulu-Natal. Other provinces in South Africa should be analyzed for ascertaining a fuller South African impact.

## Data availability statement

The original contributions presented in the study are included in the article/Supplementary material, further inquiries can be directed to the corresponding author.

## Ethics statement

The studies involving human participants were reviewed and approved by Research & Ethics Committee, UKZN. The patients/participants provided their written informed consent to participate in this study.

## Author contributions

All authors listed have made a substantial, direct, and intellectual contribution to the work. This research has been extracted from a Masters dissertation.

## Conflict of interest

The authors declare that the research was conducted in the absence of any commercial or financial relationships that could be construed as a potential conflict of interest.

## Publisher’s note

All claims expressed in this article are solely those of the authors and do not necessarily represent those of their affiliated organizations, or those of the publisher, the editors and the reviewers. Any product that may be evaluated in this article, or claim that may be made by its manufacturer, is not guaranteed or endorsed by the publisher.

## Supplementary materials

The Supplementary materials for this article can be found online at: https://www.frontiersin.org/articles/10.3389/fpsyg.2022.921343/full#supplementary-material
