# Supplementary material for: Emotional intelligence as a contributor to enhancing educators’ quality of life in the COVID-19 era
Source: Front Psychol. 2022 Aug 22;13:921343. doi: 10.3389/fpsyg.2022.921343 (PMC9443812; doi:10.3389/fpsyg.2022.921343)
Supplement: Supplementary file 1 [file Table_1.pdf]

10. Appendices

Appendix A: Cronbach Coefficient Alpha

	Number of Items	Cronbach's Alpha	Inter-Item Correlation Coefficient Means
Appraisal of Emotions	3	0.442	0.209
Regulation of Emotions	4	0.651	0.318
Utilisation of Emotions	4	0.585	0.260
Physical Health	3	0.769	0.526
Psychological Health	2	0.652	0.484
Social Relationships	3	0.795	0.564
Environmental Health	3	0.767	0.523
