# Supplementary material for: Emotional intelligence as a contributor to enhancing educators’ quality of life in the COVID-19 era
Source: Front Psychol. 2022 Aug 22;13:921343. doi: 10.3389/fpsyg.2022.921343 (PMC9443812; doi:10.3389/fpsyg.2022.921343)
Supplement: Supplementary file 2 [file Table_2.pdf]

Appendix B: Factor Analysis

	Kaiser-Meyer-Olkin Measure of Sampling Adequacy	Bartlett's Test of Sphericity		
		Approx. Chi-Square	df	Sig.
Appraisal of Emotions	0.513	10.286	3	0.016
Regulation of Emotions	0.647	58.102	6	0.000
Utilisation of Emotions	0.653	38.412	6	0.000
Physical Health	0.604	99.492	3	0.000
Psychological Health	0.500	28.126	1	0.000
Social Relationships	0.694	99.073	3	0.000
Environmental Health	0.677	85.261	3	0.000
