# Supplementary material for: Emotional intelligence as a contributor to enhancing educators’ quality of life in the COVID-19 era
Source: Front Psychol. 2022 Aug 22;13:921343. doi: 10.3389/fpsyg.2022.921343 (PMC9443812; doi:10.3389/fpsyg.2022.921343)
Supplement: Supplementary file 3 [file Table_3.pdf]

Appendix C: Emotional intelligence section analysis scoring patterns

**APPRAISAL**					
		When I am in a positive mood, solving problems is easy for me	I motivate myself by imagining a good outcome to tasks I take on	I know why my emotions change	I can tell how people are feeling by listening to the tone of their voice
Strongly Disagree	Count	1	0	2	1
	Row N %	0.9%	0.0%	1.9%	0.9%
Disagree	Count	1	2	3	4
	Row N %	0.9%	1.9%	2.8%	3.7%
Neither Agree nor Disagree	Count	4	7	16	11
	Row N %	3.7%	6.5%	14.8%	10.2%
Agree	Count	53	58	50	59
	Row N %	49.1%	53.7%	46.3%	54.6%
Strongly Agree	Count	49	41	37	33
	Row N %	45.4%	38.0%	34.3%	30.6%
**REGULATION**					
		I have control over my emotions	I seek out activities that make me happy	I expect that I will do well on most things I try	I am aware of the non-verbal messages I send to others
Strongly Disagree	Count	1	0	1	1
	Row N %	0.9%	0.0%	0.9%	0.9%
Disagree	Count	3	0	3	6
	Row N %	2.8%	0.0%	2.8%	5.6%
Neither Agree nor Disagree	Count	21	7	10	15
	Row N %	19.4%	6.5%	9.3%	13.9%
Agree	Count	54	46	54	58
	Row N %	50.0%	42.6%	50.0%	53.7%
Strongly Agree	Count	29	55	40	28
	Row N %	26.9%	50.9%	37.0%	25.9%
**UTILISATION**					
		When I am in a positive mood, I am able to come up with new ideas	When I feel a change in emotions, I tend to come up with new ideas	I recognise the emotions people are experiencing by looking at their facial expressions	I am aware of the non-verbal messages other people send
Strongly Disagree	Count	0	2	3	0
	Row N %	0.0%	1.9%	2.8%	0.0%
Disagree	Count	1	11	8	3
	Row N %	0.9%	10.2%	7.4%	2.8%
Neither Agree nor Disagree	Count	2	33	7	13
	Row N %	1.9%	30.6%	6.5%	12.0%
Agree	Count	47	47	60	66
	Row N %	43.5%	43.5%	55.6%	61.1%
Strongly Agree	Count	58	15	30	26
	Row N %	53.7%	13.9%	27.8%	24.1%
Chi Square: p-value: 0.0000					
