# Supplementary material for: Emotional intelligence as a contributor to enhancing educators’ quality of life in the COVID-19 era
Source: Front Psychol. 2022 Aug 22;13:921343. doi: 10.3389/fpsyg.2022.921343 (PMC9443812; doi:10.3389/fpsyg.2022.921343)
Supplement: Supplementary file 4 [file Table_4.pdf]

Appendix D: Quality of life section analysis scoring patterns

PHYSICAL HEALTH				
		I have enough energy for my everyday life	I am satisfied with my sleep	I am satisfied with my capacity for work
Strongly Disagree	Count	2	7	1
	Row N %	1.9%	6.5%	0.9%
Disagree	Count	13	27	4
	Row N %	12.0%	25.0%	3.7%
Neither Agree nor Disagree	Count	19	20	12
	Row N %	17.6%	18.5%	11.1%
Agree	Count	51	36	66
	Row N %	47.2%	33.3%	61.1%
Strongly Agree	Count	23	18	25
	Row N %	21.3%	16.7%	23.1%
PSYCHOLOGICAL HEALTH				
		I feel my life to be meaningful	I am able to concentrate	I often have negative feelings such as blue mood, despair, anxiety and depression
Strongly Disagree	Count	0	0	13
	Row N %	0.0%	0.0%	12.0%
Disagree	Count	0	0	26
	Row N %	0.0%	0.0%	24.1%
Neither Agree nor Disagree	Count	3	12	25
	Row N %	2.8%	11.1%	23.1%
Agree	Count	56	64	37
	Row N %	51.9%	59.3%	34.3%
Strongly Agree	Count	49	32	7
	Row N %	45.4%	29.6%	6.5%
Chi Square	p-value	0.0000	0.0000	0.0000
SOCIAL RELATIONSHIPS				
		I am satisfied with my personal relationships	I am satisfied with my sensual relationship	I am satisfied with the support I get from my friends
Strongly Disagree	Count	0	2	1
	Row N %	0.0%	1.9%	0.9%
Disagree	Count	4	3	2
	Row N %	3.7%	2.8%	1.9%
Neither Agree nor Disagree	Count	4	6	11
	Row N %	3.7%	5.6%	10.2%
Agree	Count	59	65	61
	Row N %	54.6%	60.2%	56.5%
Strongly Agree	Count	41	32	33
	Row N %	38.0%	29.6%	30.6%
ENVIRONMENTAL HEALTH				
		My physical environment is healthy	I have enough money to meet my needs	I am satisfied with my mode of transportation
Strongly Disagree	Count	1	5	4
	Row N %	0.9%	4.6%	3.7%
Disagree	Count	2	12	6

	Row N %	1.9%	11.1%	5.6%
Neither Agree nor Disagree	Count	13	17	9
	Row N %	12.0%	15.7%	8.3%
Agree	Count	59	52	50
	Row N %	54.6%	48.1%	46.3%
Strongly Agree	Count	33	22	39
	Row N %	30.6%	20.4%	36.1%
Chi Square: p-value: 0.0000				
