# Supplementary material for: Emotional intelligence as a contributor to enhancing educators’ quality of life in the COVID-19 era
Source: Front Psychol. 2022 Aug 22;13:921343. doi: 10.3389/fpsyg.2022.921343 (PMC9443812; doi:10.3389/fpsyg.2022.921343)
Supplement: Supplementary file 5 [file Table_5.pdf]

Appendix E: Pearson's Correlation Coefficient

		Appraisal	Regulation	Utilization	Physical Health	Psychological Health	Social Relationships	Environmental Health
Appraisal	Pearson Correlation	1						
	Sig. (2-tailed)							
	N	108						
Regulation	Pearson Correlation	.528**	1					
	Sig. (2-tailed)	0,000						
	N	108	108					
Utilization	Pearson Correlation	.471**	.594**	1				
	Sig. (2-tailed)	0,000	0,000					
	N	108	108	108				
Physical Health	Pearson Correlation	.375**	.393**	.255**	1			
	Sig. (2-tailed)	0,000	0,000	0,008				
	N	108	108	108	108			
Psychological Health	Pearson Correlation	.482**	.503**	.385**	.587**	1		
	Sig. (2-tailed)	0,000	0,000	0,000	0,000			
	N	108	108	108	108	108		
Social Relationships	Pearson Correlation	.397**	.358**	.217*	.444**	.637**	1	
	Sig. (2-tailed)	0,000	0,000	0,024	0,000	0,000		
	N	108	108	108	108	108	108	
Environmental Health	Pearson Correlation	.349**	.412**	.281**	.564**	.579**	.514**	1
	Sig. (2-tailed)	0,000	0,000	0,003	0,000	0,000	0,000	
	N	108	108	108	108	108	108	108
**. Correlation is significant at the 0.01 level (2-tailed).								
*. Correlation is significant at the 0.05 level (2-tailed).								
