# Supplementary material for: Emotional intelligence as a contributor to enhancing educators’ quality of life in the COVID-19 era
Source: Front Psychol. 2022 Aug 22;13:921343. doi: 10.3389/fpsyg.2022.921343 (PMC9443812; doi:10.3389/fpsyg.2022.921343)
Supplement: Supplementary file 6 [file Table_6.pdf]

Appendix F: Chi-square tests

		Gender	Age	Race	Marital Status	Your Highest Level of Completed Education	Number of Years in the Teaching Profession	Level (s) currently Teaching	Employment Status
When I am in a positive mood, solving problems is easy for me	Chi-square	10,471	17,984	4,971	13,724	31,714	21,134	59,479	7,617
	Sig.	.033*	0,325	0,761	0,619	.002*	0,173	.000*	0,107
When I am in a positive mood, I am able to come up with new ideas	Chi-square	1,260	13,124	3,785	27,064	8,013	17,214	10,621	15,234
	Sig.	0,739	0,36	0,706	.008*	0,533	0,142	0,562	.002*
When I feel a change in emotions, I tend to come up with new ideas	Chi-square	6,088	20,047	10,647	13,695	28,166	28,747	16,574	1,631
	Sig.	0,193	0,218	0,223	0,621	.005*	.026*	0,414	0,803
I motivate myself by imagining a good outcome to tasks I take on	Chi-square	2,211	5,466	3,242	9,111	6,602	15,095	7,511	4,202
	Sig.	0,53	0,941	0,778	0,693	0,678	0,236	0,822	0,24
I know why my emotions change	Chi-square	2,177	13,010	5,855	21,220	25,876	25,171	22,084	5,099
	Sig.	0,703	0,672	0,663	0,17	.011*	0,067	0,141	0,277
I have control over my emotions	Chi-square	1,448	13,064	3,797	15,387	9,318	10,917	11,107	1,854
	Sig.	0,836	0,668	0,875	0,496	0,676	0,815	0,803	0,763
I seek out activities that make me happy	Chi-square	1,458	4,576	3,358	9,704	7,937	4,847	8,688	2,500
	Sig.	0,482	0,802	0,5	0,286	0,243	0,774	0,369	0,286
I expect that I will do well on most things I try	Chi-square	3,436	19,248	11,656	13,932	13,569	10,694	16,876	2,674
	Sig.	0,488	0,256	0,167	0,604	0,329	0,828	0,394	0,614
I recognise the emotions people are experiencing by looking at their facial expressions	Chi-square	3,155	12,902	5,502	17,936	10,467	16,131	12,989	4,725
	Sig.	0,532	0,68	0,703	0,328	0,575	0,444	0,674	0,317
I can tell how people are feeling by listening to the tone of their voice	Chi-square	3,737	12,346	4,013	16,112	6,830	18,299	15,219	1,146
	Sig.	0,443	0,72	0,856	0,445	0,869	0,307	0,509	0,887
I am aware of the non-verbal messages I send to others	Chi-square	7,401	10,717	5,652	9,753	13,481	19,791	16,788	3,454
	Sig.	0,116	0,827	0,686	0,879	0,335	0,23	0,399	0,485
I am aware of the non-verbal messages other people send	Chi-square	3,120	4,609	8,984	14,271	9,195	12,862	11,794	7,970
	Sig.	0,374	0,97	0,175	0,284	0,419	0,379	0,462	.047*
I have enough energy for my everyday life	Chi-square	1,736	23,979	8,286	8,818	16,130	14,689	22,337	4,456
	Sig.	0,784	0,09	0,406	0,921	0,185	0,548	0,133	0,348
I am satisfied with my sleep	Chi-square	2,005	7,171	6,914	10,219	22,569	8,666	9,709	2,181
	Sig.	0,735	0,97	0,546	0,855	.032*	0,927	0,881	0,703
I am satisfied with my capacity for work	Chi-square	10,387	9,937	8,847	12,219	13,966	12,913	8,404	6,892
	Sig.	.034*	0,87	0,355	0,729	0,303	0,679	0,936	0,142
I feel my life to be meaningful	Chi-square	1,860	7,813	2,319	15,782	5,777	2,666	6,628	9,698
	Sig.	0,395	0,452	0,677	.046*	0,449	0,954	0,577	.008*
I am able to concentrate	Chi-square	0,906	6,167	5,119	6,383	5,963	9,717	8,387	6,945
	Sig.	0,636	0,629	0,275	0,604	0,427	0,285	0,397	.031*
I often have negative feelings such as blue mood, despair, anxiety and depression	Chi-square	2,766	12,491	19,024	18,887	7,816	14,287	7,953	2,169
	Sig.	0,598	0,71	.015*	0,275	0,799	0,577	0,95	0,705

I am satisfied with my personal relationships	Chi-square	1,560	17,171	5,736	20,073	12,960	19,181	11,958	51,923
	Sig.	0,669	0,143	0,453	0,066	0,164	0,084	0,449	.000*
I am satisfied with my sensual relationship	Chi-square	2,571	12,174	4,975	41,583	9,517	40,598	10,859	57,028
	Sig.	0,632	0,732	0,76	.000*	0,658	.001*	0,818	.000*
I am satisfied with the support I get from my friends	Chi-square	10,994	11,612	4,583	7,178	18,054	18,488	10,484	7,881
	Sig.	.027*	0,77	0,801	0,97	0,114	0,296	0,84	0,096
My physical environment is healthy	Chi-square	2,428	17,138	13,430	15,315	14,474	31,019	14,301	2,280
	Sig.	0,658	0,377	0,098	0,502	0,271	.013*	0,576	0,684
I have enough money to meet my needs	Chi-square	3,802	19,604	11,885	29,429	12,929	25,947	17,118	8,590
	Sig.	0,433	0,239	0,156	.021*	0,374	0,055	0,378	0,072
I am satisfied with my mode of transportation	Chi-square	4,403	16,405	20,098	15,288	16,542	20,166	7,081	18,215
	Sig.	0,354	0,425	.010*	0,504	0,168	0,213	0,972	.001*
*. Correlation is significant at the 0.05 level (2-tailed).									
