# Supplementary figures and images for: Emotional intelligence as a contributor to enhancing educators’ quality of life in the COVID-19 era
Source: Front Psychol. 2022 Aug 22;13:921343. doi: 10.3389/fpsyg.2022.921343 (PMC9443812; doi:10.3389/fpsyg.2022.921343)

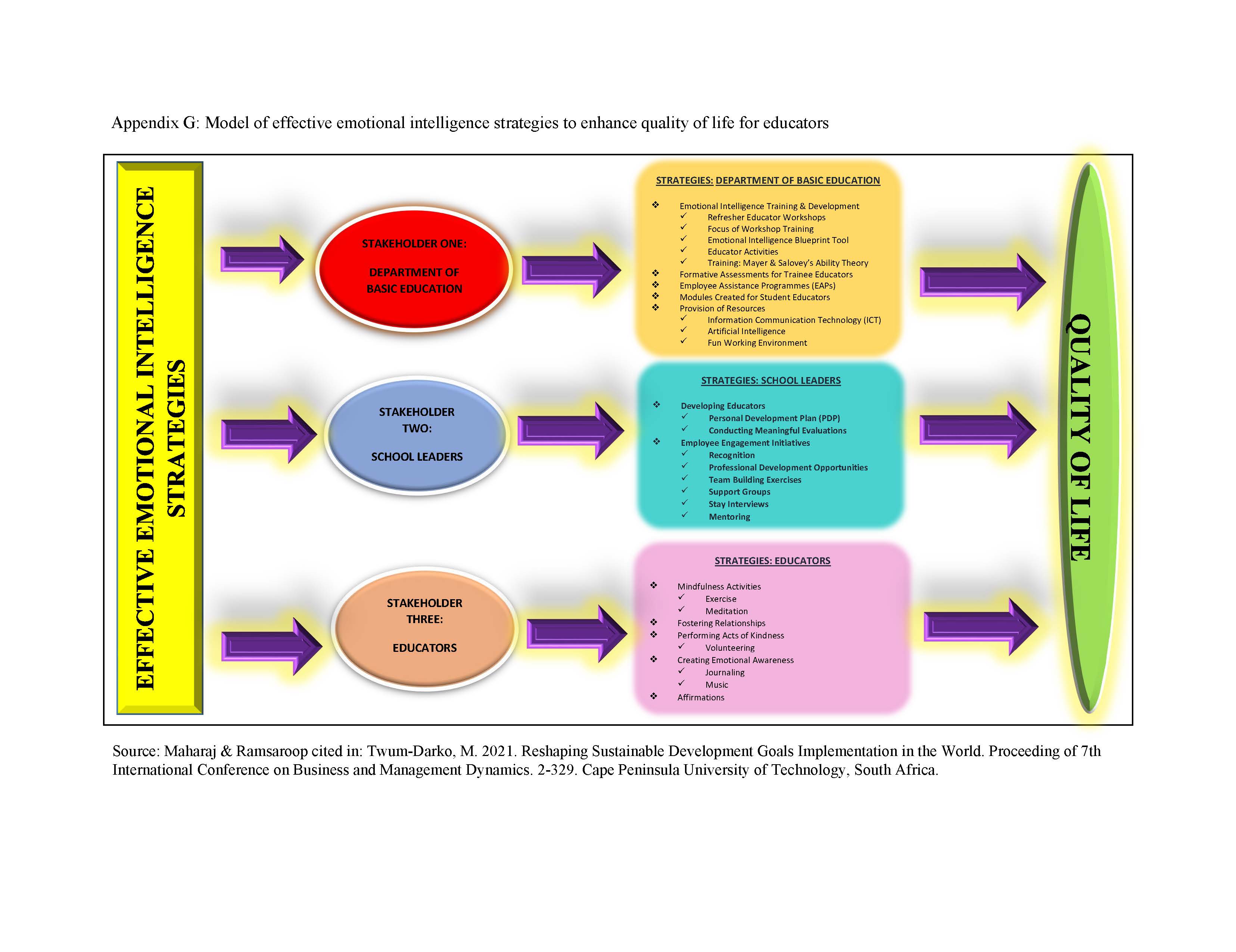

Supplement: Supplementary file 7 [file Image_1.jpg]
